# Effects of Comparative Killing by Pradofloxacin and Seven Other Antimicrobials Against Varying Bacterial Densities of Swine Isolates of *Pasteurella multocida*

**DOI:** 10.3390/microorganisms13020221

**Published:** 2025-01-21

**Authors:** Joseph M. Blondeau, Shantelle D. Fitch

**Affiliations:** 1 Department of Clinical Microbiology, Royal University Hospital and Saskatchewan Health Authority, Saskatoon, SK S7N 0W8, Canada; shantelle.fitch@saskhealthauthority.ca; 2 Pathology and Laboratory Medicine and Ophthalmology, Departments of Microbiology and Immunology, University of Saskatchewan, Saskatoon, SK S7N 0W8, Canada

**Keywords:** pradofloxacin, swine respiratory disease, *P. multocida*, killing, therapeutic drug concentration, bactericidal

## Abstract

Bacterial killing is important for recovering from infection. *Pasteurella multocida* is a key bacterial pathogen causing swine respiratory disease and is associated with substantial mortality. Antimicrobial therapy remains an important therapeutic intervention for treating infected animals. Pradofloxacin (fluoroquinolone) is the most recently approved antimicrobial agent for treating pigs with swine respiratory disease. We compared in vitro killing of swine *P. multocida* strains by pradofloxacin in comparison to ceftiofur, enrofloxacin, florfenicol, marbofloxacin, tildipirosin, tilmicosin, and tulathromycin over a range of bacterial densities and four clinically relevant drug concentrations. Pradofloxacin killed 92–96.9% of cells across 10^6^–10^8^ cfu/mL densities at the mutant prevention drug concentration following 2–24 h of drug exposure, 96.9–98.9% of cells across 10^6^–10^9^ cfu/mL at the maximum serum drug concentration following 30 min of drug exposure, increasing to 99.9–100% kill following 12–24 h of drug exposure. At the maximum tissue drug concentration and against bacterial densities of 10^6^–10^9^ cfu/mL, pradofloxacin killed 91.3–99.8% of cells following 2 h of drug exposure, which increased to 99.9–100% kill following 12–24 h of drug exposure. Pradofloxacin was rapidly bactericidal across a range of bacterial densities and at clinically relevant drug concentrations. Pradofloxacin will be an important antibiotic for treating pigs with swine respiratory disease and where clinically indicated.

## 1. Introduction

Swine respiratory disease is due to bacterial infection; however, when viruses are involved in the pathogenesis, it is referred to as porcine respiratory disease complex. The interaction between viral infection and immunity, leading to disruption in normal immune functioning, predisposes animals to secondary bacterial infections, giving rise to bacterial pneumonia. Management and environmental factors impacting this complex disease include overcrowding, mixing different sources of animals, continuous flow ventilation, high gilt replacement rate, and temperature fluctuations. *Pasteurella multocida* is an opportunistic bacterial pathogen associated with swine respiratory disease and may cause atrophic rhinitis and pneumonia [1]. *P. multocida* has numerous virulence factors including dermonecrotic toxin, adhesions, outer membrane protein toxins, fimbriae, iron acquisition proteins, capsules, and lipopolysaccharides [1]. Antimicrobial resistance is also considered as a virulence factor.

In vitro determination of susceptibility or resistance is by minimum inhibitory concentration (MIC), which is based on testing 10^5^ cfu/mL of bacteria against doubling dilutions of drug. Bacterial densities fluctuate during infection [2] and densities may exceed the number necessary for spontaneous mutation to occur (1 × 10^−7^–1 × 10^−9^) [3,4]. As such, spontaneous mutant cells may arise during antimicrobial therapy and where mutant cells are not inhibited in the presence of the drug [5]. The mutant prevention concentration (MPC) is an in vitro assay utilizing ≥10^9^ CFUs applied to drug-containing agar plates to determine the drug concentration blocking growth of the least susceptible cells (including spontaneous mutants) present in high-density bacterial populations. Higher-density bacterial populations are known to exist during infection of the central nervous system [6,7], respiratory tract [8,9], urinary tract [10,11], and skin and soft tissue [12]. A third in vitro measurement is the killing of bacteria by antimicrobial agents at clinically relevant drug concentrations [13]. Measurements of kill over a range of bacterial densities provide insight on bug–drug interaction with varied densities of organisms.

Pradofloxacin is the newest veterinary fluoroquinolone to be approved for use in food animals and specifically in swine for swine respiratory disease. Pradofloxacin is characterized as a third-generation dual-targeting compound with simultaneous activity against the enzyme targets DNA gyrase and topoisomerase IV in bacteria [14,15]. Dual or multi-targeting is argued to reduce the likelihood for resistance selection, as an organism would be required to possess two or more resistance mutations for growth in the presence of the drug [16]. In this study, we measured the in vitro killing of swine isolates of *P. multocida* by pradofloxacin in comparison to ceftiofur, enrofloxacin, florfenicol, marbofloxacin, tildipirosin, tilmicosin, and tulathromycin using four clinically relevant drug concentration for each drug: the measured MIC and MPC, maximum serum, and maximum tissue drug concentrations. Additionally, killing was measured against varying bacterial densities ranging 10^6^–10^9^ cfu/mL. Pradofloxacin showed bactericidal killing between 96–>99% of bacterial cells following 30 min to 4 h of drug exposure at the maximum serum drug concentration and against all bacterial densities tested. Similar results were seen with killing at the maximum tissue drug concentration.

## 2. Materials and Methods

### 2.1. Bacterial Strains

Three unique *P. multocida* isolates collected from swine in the USA were used and provided with compliments of Purdue University (West Lafayette, IN, USA). Organism identification was confirmed by matrix-assisted laser desorption ionization–time of flight (MALDI-TOF) (BioMerieux, St. Laurent, QC, Canada) and confirmed by Vitek II (BioMerieux, St. Laurent, QC, Canada). Isolates were cultured on tryptic soy agar containing 5% sheep red blood cells (BA) (Oxoid, Nepean, ON, Canada) in oxygen (O_2_) at 35–37 °C for 18–24 h. Single colonies were picked with a sterile wooden stick (6″ applicator stick, Fisherbrand, ThermoFisher Scientific, Mississauga, ON, Canada) transferred to cryovials (Corning Incorporated, Corning, NY, USA) containing skim milk (Hardy Diagnostic, Santa Maria, CA, USA) and stored at −70 °C. Each selected isolate included in this study had to be susceptible to all antimicrobials based on currently available recommended susceptibility MIC breakpoints [17].

### 2.2. Antimicrobial Compounds

Enrofloxacin (99% pure) and pradofloxacin (99% pure) were obtained from Elanco (Greenfield, IN, USA) and prepared as per the manufacturers’ instructions. Injection-grade ceftiofur (Zoetis, Kirkland QC, Canada), florfenicol (Merck, Kirkland, QC, Canada) marbofloxacin (Vetoquinol, Laval Trie QC, Canada), tildipirosin (Merck Kirkland, QC, Canada), tilmicosin, and tulathromycin (Zoetis, Kirkland, QC, Canada) were purchased commercially and prepared in accordance with the manufacturer’s directions. Fresh stock solutions or samples stored at −70 °C were used for each experiment.

### 2.3. MIC Testing

MIC testing was performed as per the Clinical and Laboratory Standards Institute recommended procedure [18]. Isolates were thawed at room temperature and subcultured on ×2 BA, incubated in 0_2_ for 18–24 h at 35–37 °C and examined for isolated colonies and purity. Two-fold drug concentration increments of each drug individually were added to 96-well microdilution trays in Mueller–Hinton broth (MHB) (Difco Laboratories, Detroit, MI, USA). Suspensions of *P. multocida* (0.5 McFarland standard) were diluted to a final inoculum of 5 × 10^5^ cfu/mL, added to microtiter trays, and incubated for 18–24 h at 35–37 °C in 0_2_, and the lowest drug concentration preventing visible bacterial growth was the MIC. American Type Culture Collection (ATCC) control strains were included in each MIC assay to ensure assays were within acceptable quality control performance ranges. These included *Enterococcus faecalis* 29212, *Escherichia coli* 25922, *Staphylococcus aureus* 29213, and *Pseudomonas aeruginosa* 27853.

### 2.4. MPC Testing

A total of 5 BA plates per isolate was inoculated to produce a lawn of confluent growth and incubated for 18–24 h in O_2_ at 35–37 °C, following which the complete plate contents of bacterial growth were removed from each plate with sterile swabs, transferred to 100 mL of MHB and then incubated in O_2_ for 18–24 h at 35–37 C in O_2_ [19,20]. Bacterial densities of >3 × 10^9^ cfu/mL were determined by spectrophotometric readings (600 nm) >0.3 (Thermo Scientific Genesys 10s vis, Mississauga, ON, Canada) and colony counts. A bacterial inoculum of >10^9^ cfu in 100 µL was applied to BA plates containing an individual antimicrobial drug over a range of concentrations from one dilution below the measured MIC to the determined MPC value. In-house-prepared drug-containing agar plates were used within 7 days of preparation. Inoculated plates were incubated (as described) and examined at 24 and 48 h for bacterial colony growth. The MPC was the lowest drug concentration preventing growth following 48 h incubation. The ATCC control strains previously described were included in each experiment.

### 2.5. Kill Experiments

We have previously described the method for the kill experiments [13]. *P. multocida* isolates were incubated in 0_2_ at 35–37 °C in O_2_ for 18–24 h on BA; an inoculum was subsequently transferred to MHB, incubated at 35–37 °C in O_2_ for 2 h, and spectrophotometric readings of ≥0.3 verified cell densities ≥10^9^ cells/mL [19]. Inocula were further adjusted to achieve cell densities of 10^6^–10^9^ cfu/mL in MHB for each drug individually (ceftiofur, enrofloxacin, florfenicol, marbofloxacin, pradofloxacin, tildipirosin, tilmicosin, and tulathromycin), added in concentrations based on the determined MIC and MPC or pre-established maximum serum or tissue drug concentrations for each molecule [21]. At each time point, three aliquots of each resulting solution were applied to drug-free BA plates and incubated as described [16,17]. Measurements of antimicrobial killing (log_10_ reduction in viable cells and percentage of cells killed) were obtained before (time 0) and at 30 min and 1, 2, 4, 6, 12, and 24 h of drug exposure. Means of the results for each of the 3 aliquots were calculated. Each time point represents 9 individual measurements (3 aliquots × 3 individual strains).

### 2.6. Statistical Analysis

Statistical analysis of the data was performed by means of a repeated-measures ANCOVA for each drug/log-exposure dataset, with fixed effects consisting of drug, exposure time, and drug-by-time interaction. In each model, CFU count at time 0 was included as a covariate and a compound symmetric covariance structure was used. The CFU counts were logarithmically transformed to achieve a normal distribution. Bonferroni adjustments for multiple comparisons were made. Values of *p* ≤ 0.05 were considered significant for all analyses list the authority that provided approval and the corresponding ethical approval code. *p* value ≤ to 0.05 was considered significant.

We investigated the relationship between dependence and time. For this, we fitted a repeated measures model with covariate (Time0), treatment, time, and treatment-by-time interactions as fixed effects. The time factor in the model measures whether the mean response differs over time. Also, the treatment-by-time interaction term tests whether the groups’ mean responses are changing over time. A significant change over time for these factors was declared if *p*-value < 0.05. *p* value ≤ to 0.05 was considered significant.

## 3. Results

The determined MIC and MPC values along with drug concentrations used in the kill assays are summarized in Table 1.

### 3.1. Pasteurella Multocida

#### 3.1.1. Minimum Inhibitory Concentration (MIC)

Exposure of 10^6^ cfu/mL of *P. multocida* to the MIC drug concentrations of the eight antimicrobial agents tested yielded the following results (Table 2): Pradofloxacin killed 11.2–26.9% of cells following 30 min to 2 h of drug exposure, 97.7% following 4 h and 98.8% following 24 h. By comparison, marbofloxacin killed up to 8.7% of cells following 2 h of drug exposure, 90.5% following 4 h, and 9.6% after 24 h of drug exposure. Tildipirosin killed 34.8–49.1% of cells following 4–6 h of drug exposure and 88.1% and 90.5% following 12 and 24 h of drug exposure. For ceftiofur, between 15.7% to 72.2% of cells were killed following 30 min to 6 h of drug exposure, following which regrowth occurred. Minimal or no killing occurred in the presence of the other drugs. Statistically significant differences in kill rate were not seen between any agents following 30 min, 1, 2, 4, 6, and 12 h of drug exposure. Following 24 h of drug exposure, pradofloxacin (4.1 log_10_, 98.8% kill) killed more cells than ceftiofur (growth, *p* < 0.001), enrofloxacin (growth, *p* < 0.0001), tildipirosin (1.2 log_10_, 90.5% kill, *p* = 0.0002), tilmicosin (growth, *p* ≤ 0.0001), and tulathromycin (growth, *p* < 0.0001). Regarding time, significant differences were seen in treatment (*p* = 0.0217), time (*p* < 0.0001), and treatment by time (*p* < 0.0001).

Exposure of 10^7^ cfu/mL of *P. multocida* to the MIC drug concentrations did not result in any significant differences in kill rates between the drug tested following 30 min, 1, 2, 4, 6, and 12 h of drug exposure (Table 3). Pradofloxacin killed up to 87.8% of cells following 12 h for drug exposure as compared to up to 49.4% by ceftiofur following 6 h of drug exposure. Minimal or no killing occurred in the presence of the other agents, and growth or regrowth occurred with all drugs. Following 24 h of drug exposure, pradofloxacin (4.1 log_10_) killed more cells than ceftiofur (growth, *p* < 0.0001), enrofloxacin (growth, *p* < 0.0001), marbofloxacin (growth, *p* < 0.0001), tildipirosin (1.2 log_10_, *p* = 0.0003), tilmicosin (growth, *p* < 0.0001), and tulathromycin (growth, *p* < 0.0001). For time, statistically significant differences were seen for treatment (*p* = 0.0380) and treatment by time (*p* < 0.001).

Exposure of 10^8^ cfu/mL of *P. multocida* to the MIC drug concentrations did not result in significant differences in kill rates between the drugs following 30 min, 1, 2, 4, 6, and 12 h of drug exposure (Table 4). Pradofloxacin killed between 43.6 and 60.5% of cells following 30 min to 4 h of drug exposure. Minimal or no killing was seen with the other agents over these same time points. Following 24 h of drug exposure, pradofloxacin (1.6 log_10_) killed more cells than ceftiofur (growth, *p* = 0.0189), florfenicol (growth, *p* = 0.0272), marbofloxacin (growth, *p* = 0.0005), tildipirosin (growth, *p* = 0.0015), tilmicosin (0.04 log_10_, *p* = 0.0354), and tulathromycin (growth, *p* = 0.0066). For time, a statistically significant difference was seen for treatment by time (*p* = 0.0010).

Exposure of 10^9^ cfu/mL of *P. multocida* to the MIC drug concentrations did not result in statistically significant differences in kill rates between the drugs following 30 min, 1, 2, 4, 6, 12, and 24 h of drug exposure (Table 5). Killing was minimal by all agents with killing between growth to 31.9% kill following 24 h of drug exposure. No statistically significant differences were seen for treatment by time.

#### 3.1.2. Mutant Prevention Concentration (MPC)

Exposure of 10^6^ cfu/mL of MPC drug concentrations of the eight antimicrobial agents tested yielded the following results (Table 2): Pradofloxacin killed 56.7% of cells following 30 min of drug exposure, which increased to 96.9% following 4 h and 99.9% following 24 h of drug exposure. For enrofloxacin and marbofloxacin, growth to 41.9% kill was seen following 30 min of drug exposure, which increased to 89.5–90.9% after 4 h and 70.4–99.8% after 24 h of drug exposure. Ceftiofur killed the following percentage of cells following 30 min, 4 h, and 24 h of drug exposure: 8.6%, 75.8%, and 99.6%. Florfenicol killed up to 53.8% of cells following 6 h of drug exposure but growth occurred thereafter. For tildipirosin, tilmicosin, and tulathromycin, the following percentages of cells were killed following 30 min, 4 h, and 24 after of drug exposure: growth to 31.4%, 32.7–82.6%, and 99.4–99.9%. Statistically significant differences in kill rates were not seen between agents following 30 min, 1, 2, 4, 6, and 12 h of drug exposure. Following 24 h of drug exposure, ceftiofur (3.7 log_10_, 99.6% kill) killed more cells than enrofloxacin (0.8 log_10_, 70.4% kill, *p* = 0.0514). Pradofloxacin (5.2 log_10_, 99.9% kill) killed more cells than enrofloxacin (*p* < 0.0001), florfenicol (0.9 log_10_, *p* < 0.0001), and tulathromycin (2.3 log_10_, 99.4% kill, *p* = 0.0527). Marbofloxacin (4.8 log_10_, 99.8% kill) killed more cells than florfenicol (*p* = 0.0009) and enrofloxacin (*p* = 0.0001). Statistically significant differences were seen for treatment (*p* = 0.0413), time (*p* < 0.0001) and treatment by time (*p* < 0.0001).

Following exposure of 10^7^ cfu/mL to the MPC drug concentrations, statistically significant differences in kill between the agents were not seen following 30 min, 1, 2, 4, 6, and 12 h of drug exposure (Table 3). Pradofloxacin killed 65.0% of cells following 30 min of drug exposure and increasing to 97.9% after 4 h and 99.9% after 24 h of drug exposure. The kill values, respectively, for enrofloxacin and marbofloxacin for the same time points were as follows: 27.5% and 65.3%, 77.6 and 45.9, and growth to 14.2%. Tulathromycin killed 39.7 and 36.9% of cells following 4 and 24 h of drug exposure. Tildipirosin killed 5.7%, 56.3%, and 99.6% of cells following 30 min, 4 h, and 24 h of drug exposure. Tilmicosin killed 64.3 and 61.7% of cells following 4 and 24 h of drug exposure. Following 24 h of drug exposure, pradofloxacin (4.6 log_10_, 99.9% kill) killed more cells than ceftiofur (1.0 log_10_, 89.7% kill, *p* = 0.0111), florfenicol (growth, *p* = 0.0002), and tulathromycin (1.0 log_10_, 36.9% kill). Statistically significant differences were seen for time (*p* < 0.0001) and treatment by time (*p* = 0.0091).

Following exposure of 10^8^ and 10^9^ cfu/mL of *P. multocida* to the MPC drug concentrations, statistically significant differences in kill rates were not seen between the agents tested at any time point following drug exposure. For the 10^8^ cfu/mL experiments, pradofloxacin killed 65.9%, 96.3%, and 96.9% of cells following 30 min, 4 h, and 24 h of drug exposure (Table 4). Enrofloxacin killed up to 48.0% of cells following 1 h of drug exposure but killing declined thereafter. Similarly, marbofloxacin killed up to 72.2% following 2 h of drug exposure but killing declined thereafter such that by 24 h after drug exposure, 36.9% of cells were killed. Ceftiofur killed up to 47.5% of cells after 6 h of drug exposure, which decreased to 25.5% kill after 24 h of drug exposure. Growth occurred in the presence of florfenicol and tulathromycin over all time points, and for tildipirosin and tilmicosin, 30.3–37.8% of cells were killed following 24 h of drug exposure and not more at any earlier time point. A statistically significant difference was seen for time (*p* < 0.0001).

For the 10^9^ cfu/mL kill measurements, pradofloxacin killed 60.9%, 75.2%, and 83.4% of cells following 30 min, 6 h, and 24 h of drug exposure (Table 5). Growth to up to 44.8% kill occurred in the presence of enrofloxacin, florfenicol, marbofloxacin, and tildipirosin. For ceftiofur, 2.9%, 40.3%, and 78.8% of cells were killed following 30 min, 6 h, and 24 h of drug exposure. Growth occurred in the presence of tulathromycin over all time points, and tilmicosin killed up to 58.4% of cells following 6 h of drug exposure but killing declined thereafter such that 38.6% of cells were killed following 24 h of drug exposure. A statistically significant difference was seen for time (*p* = 0.0005).

#### 3.1.3. Maximum Serum Drug Concentration (C_max_)

Following exposure of 10^6^ cfu/mL of *P. multocida* to the maximum serum drug concentrations of the drugs tested did not result in significant differences in kill rates between the drugs following 30 min of drug exposure (Table 2). Pradofloxacin killed 98.8%, 99.9%, and 100% of cells following 30 min, 4 h, and 12 h for drug exposure; for enrofloxacin and marbofloxacin, those values, respectively, were 81.7% and 98.9%, 98.7% and 99.4%, and 100% and 99.9%. Ceftiofur killed 55.6% of cells following 30 min of drug exposure, which increased to 97.7% kill after 4 h and 99.99% kill after 24 h of drug exposure; those values for florfenicol at the same time points were growth 83.8% and 99.9%. Growth occurred in the presence of tilmicosin at all time points. Tulathromycin killed 66.5% and 93.8% of cells following 12 and 24 h of drug exposure and tildipirosin killed 40.5%, 99.3%, and 99.99% of cells following 6, 12, and 24 h of drug exposure. Following 1 h of drug exposure, marbofloxacin (2.5 log_10_, 98.9% kill) killed more cells than florfenicol (0.1 log_10_, 14.3% kill, *p* = 0.0361), tildipirosin (growth, *p* = 0.016), tilmicosin (growth, *p* = 0.0088), and tulathromycin (growth, *p* = 0.0118). Following 2 h of drug exposure, marbofloxacin (2.6 log_10_, 99.3% kill) killed more cells than florfenicol (0.2 log_10_, 16.7% kill, *p* = 0.0343), tildipirosin (growth, *p* = 0.0068), tilmicosin (growth, *p* = 0.0007), and tulathromycin (growth, *p* = 0.0017). Pradofloxacin (2.2 log_10_, 99.2% kill) killed more cells than tilmicosin (*p* = 0.0092) and tulathromycin (*p* = 0.0309). Following 4 h of drug exposure, ceftiofur (2.0 log_10_, 97.7% kill) killed more cells than tilmicosin (growth, *p* = 0.0249). Enrofloxacin (2.1 log_10_, 98.7% kill) killed more cells than tilmicosin (*p* = 0.0162). Marbofloxacin (2.6 log_10_, 99.4% kill) killed more cells than tildipirosin (0.3 log_10_, 37.1% kill, *p* = 0.0038), tilmicosin (growth *p* < 0.0001), and tulathromycin (growth, *p* = 0.0002). Pradofloxacin (3.4 log_10_, 99.9% kill) killed more cells than florfenicol (0.9 log_10_, 83.8% kill, *p* = 0.0344), tildipirosin (*p* = 0.0001), tilmicosin (*p* < 0.0001), and tulathromycin (*p* < 0.0001). Following 6 h of drug exposure, ceftiofur (2.8 log_10_, 99.7% kill) killed more cells than tildipirosin (0.3 log_10_, 40.5% kill, *p* = 0.026), tilmicosin (growth, *p* < 0.0001), and tulathromycin (growth, *p* = 0.0084). Enrofloxacin (2.4 log_10_, 99.6% kill) killed more cells than tilmicosin (*p* = 0.0004). Marbofloxacin (2.7 log_10_, 99.9% kill) killed more cells than tildipirosin (*p* = 0.0219), tilmicosin (*p* < 0.0001), and tulathromycin (*p* = 0.0067). Pradofloxacin (3.9 log_10_, 99.9% kill) killed more cells than florfenicol (0.9 log_10_, 85.5% kill, *p* = 0.0006), tildipirosin (*p* < 0.0001), tilmicosin (*p* < 0.0001), and tulathromycin (*p* < 0.0001). Following 12 h of drug exposure, ceftiofur (4.3 log_10_, 99.9% kill) killed more cells than tilmicosin (growth, *p* < 0.0001) and tulathromycin (0.06 log_10_, 66.5% kill). Florfenicol (1.1 log_10_, 79.6% kill) killed more cells than tilmicosin (*p* = 0.0132). Marbofloxacin (3.3 log_10_, 99.8%) killed more cells than tilmicosin (*p* < 0.0001) and tulathromycin (*p* = 0.0027). Pradofloxacin (5.9 log_10_, 100% kill) killed more cells than florfenicol (*p* < 0.0001), tildipirosin (2.5 log_10_, 99.3% kill, *p* < 0.0001), tilmicosin (*p* < 0.0001), and tulathromycin *p* < 0.0001). Tildipirosin killed more cells than tilmicosin (*p* = 0.0001). Following 24 h of drug exposure, ceftiofur (5.4 log_10_, 99.99% kill) killed more cells than tilmicosin (growth, *p* < 0.0001) and tulathromycin (1.9 log_10_, 93.8% kill, *p* < 0.0001). Enrofloxacin (6.6 log_10_, 100% kill) killed more cells than florfenicol (4.0 log_10_, 99.9% kill, *p* = 0.0086), tilmicosin (*p* < 0.0001), and tulathromycin (*p* < 0.0001). Marbofloxacin (6.5 log_10_, 99.9% kill) killed more cells than florfenicol (*p* = 0.0377), tilmicosin (*p* < 0.0001), and tulathromycin (*p* < 0.0001). Pradofloxacin (6.7 log_10_, 100% kill) killed more bacteria than florfenicol (*p* = 0.0048), tilmicosin (*p* < 0.0001) and tulathromycin (*p* < 0.0001). Tildipirosin (4.9 log_10_, 99.99% kill) killed more cells than tilmicosin (*p* < 0.0001). Statistically significant differences were seen for treatment, time, and treatment by time (*p* values <0.0001) for all comparisons.

Following exposure of 10^7^ cfu/mL of *P. multocida* to the maximum serum concentrations of the drugs tested, statistically significant differences in kill rates were not seen between the agents following 30 min, 1, and 2 h of drug exposure (Table 3). Pradofloxacin killed 98.9%, 99.8%, and 100% of cells following 30 min, 4 h, and 24 after drug exposure; for enrofloxacin and marbofloxacin, those values were 83.4% and 98.5%, 99.5% and 99.45, and 99.8 and 100%. Ceftiofur killed 30.8, 94.3, and 92.4% of cells following 30 min, 4 h, and 24 h of drug exposure. Minimal killing (17.9%) but mostly growth occurred in the presence of tildipirosin, tilmicosin, and tulathromycin. Florfenicol killed 75.8% and 95.9% of cells following 12 and 24 h of drug exposure. Following 4 h of drug exposure, marbofloxacin (2.7 log_10_, 99.4% kill) killed more cells than tulathromycin (growth, *p* = 0.0485). Pradofloxacin (2.8 log_10_, 99.8% kill) killed more cells than tilmicosin (growth, *p* = 0.0377) and tulathromycin (growth, *p* = 0.0211) and approached a significant difference with tildipirosin (growth, *p* = 0.0575). Following 6 h of drug exposure, marbofloxacin (2.9 log_10_, 99.7% kill) killed more cells than tildipirosin (growth, *p* = 0.0150), tilmicosin (growth, *p* = 0.0354), and tulathromycin (*p* = 0.0060). Pradofloxacin (2.9 log_10_, 99.8% kill) killed more cells than tildipirosin (*p* = 0.0065), tilmicosin (*p* = 0.0093), and tulathromycin (*p* = 0.0027). Following 12 h of drug exposure, marbofloxacin (4.7 log_10_, 99.99% kill) killed more cells than dd ceftiofur (1.5 log_10_, 93.5% kill, *p* = 0.017), florfenicol (0.7 log_10_, 75.8% kill, *p* < 0.0001), tildipirosin (0.3 log_10_, 13% kill, *p* < 0.0001), tilmicosin (growth, *p* < 0.0001), and tulathromycin (growth, *p* < 0.000). Enrofloxacin (2.9 log_10_, 99.8% kill) killed more cells than tilmicosin (*p* = 0.0313) and tulathromycin (*p* = 0.0003). Pradofloxacin (4.9 log_10_, 99.9% kill) killed more cells than ceftiofur (*p* = 0.0017), florfenicol (*p* < 0.0001), tildipirosin (*p* < 0.0001), tilmicosin (*p* < 0.0001), and tulathromycin. Following 24 h of drug exposure, enrofloxacin (1.6 log_10_) killed more cells than ceftiofur (2.2 log_10_, 93.4% kill, *p* < 0.0001), florfenicol (1.7 log_10_, 95.9% kill, *p* < 0.0001), tildipirosin (growth, *p* < 0.0001), tilmicosin (growth, *p* < 0.0001), and tulathromycin (growth, *p* < 0.0001). Marbofloxacin (7.0 log_10_, 100% kill) killed more cells than ceftiofur (*p* < 0.0001), florfenicol (*p* < 0.0001), tildipirosin (*p* < 0.0001), tilmicosin (*p* < 0.0001), and tulathromycin (*p* < 0.0001). Pradofloxacin (7.8 log_10_, 100% kill) killed more cells than ceftiofur (*p* < 0.0001), florfenicol (*p* < 0.0001), tildipirosin (*p* < 0.0001), tilmicosin (*p* < 0.0001), and tulathromycin (*p* < 0.0001). Statistically significant differences were seen for treatment, time, and treatment by time (*p* values <0.0001) for all comparisons.

Following exposure of 10^8^ cfu/mL of *P. multocida* to the maximum serum drug concentration of the eight drugs tested, statistically significant differences in kill rates between the drugs following 30 min, 1, and 2 h of drug exposure (Table 4). Pradofloxacin killed 98.6%, 99.9%, and 99.99% of cells following 30 min, 4 h, and 24 h of drug exposure; those values for enrofloxacin and marbofloxacin were 66.1 and 98.4, 96.1 and 99.9, and 99.8 and 99.9. For ceftiofur, 55.5%, 93.1%, and 96.0% of cells were killed following 30 min, 4 h, and 23 h of drug exposure. In the presence of florfenicol, tildipirosin, tilmicosin, and tulathromycin, the maximum kill was 47.8% (tildipirosin) following 12 h of drug exposure; however, growth occurred in the presence of all drugs at most time points. Following 4 h of drug exposure, marbofloxacin (3.5 log_10_, 99.9% kill) killed more cells than tildipirosin (growth, *p* = 0.0404). Following 6 h of drug exposure, pradofloxacin (3.4 log_10_, 99.9% kill) killed more cells than tildipirosin (growth, *p* = 0.0347). Following 12 h of drug exposure, pradofloxacin (5.8 log_10_, 99.99% kill) killed more cells than ceftiofur (0.9 log_10_, 84.9% kill, *p* = 0.0002), florfenicol (growth *p* < 0.0001), tildipirosin (growth, *p* < 0.0001), tilmicosin (growth, *p* < 0.0001), and tulathromycin (*p* < 0.0001). Following 24 h of drug exposure, pradofloxacin (5.9 log_10_, 99.99% kill) killed more cells than ceftiofur (1.7 log_10_, 96.0% kill, *p* = 0.0004), florfenicol (growth, *p* < 0.0001), tildipirosin (0.1 log_10_, 16.7% kill, *p* < 0.0001), and tilmicosin (growth, *p* < 0.0001). Marbofloxacin (4.1 log_10_, 99.9% kill) killed more cells than florfenicol (*p* = 0.0053), tildipirosin (*p* = 0.0082), and tilmicosin (*p* = 0.0127). Statistically significant differences were seen for treatment, time, and treatment by time (*p* values from 0.0018–<0.0001) for all comparisons.

Following exposure of 10^9^ cfu/mL of *P. multocida* to the maximum serum drug concentrations of the eight drugs tested, statistically significant differences in kill rates were not seen between the agents following 30 min, 1, 2, and 4 h following drug exposure (Table 5). Pradofloxacin killed 96.9% of cells following 30 min of drug exposure, which increased to 99.3% and 99.99% following 4 h and 24 h of drug exposure; for enrofloxacin and marbofloxacin, those values were, respectively, 84.9% and 95.7%, 98.2% and 98.4%, and 98.6% and 98.9%. Ceftiofur and florfenicol killed the following percentages of cells following 30 min, 4 h, and 24 h of drug exposure: 7.8% and 1%, 27.3% and 23.7%, and 95.6% and 63.5%. Growth occurred at all time points in the presence of tilmicosin, and 42.2% kill occurred following 24 h exposure to tildipirosin. Up to 46.3% kill occurred in the presence of tulathromycin following 12 h of drug exposure but regrowth occurred thereafter. Following 6 h of drug exposure, pradofloxacin (3.0 log_10_, 99.7% kill) killed more cells than did tildipirosin (growth, *p* = 0.0245), tilmicosin (growth, *p* = 0.0191), and tulathromycin (growth, *p* = 0.0498). Following 12 h of drug exposure, pradofloxacin (3.0 log_10_, 99.9% kill) killed more cells than florfenicol (growth, *p* = 0.0002), tildipirosin (growth, *p* = 0.0005), tilmicosin (growth, *p* = 0.0002), and tulathromycin (growth, *p* = 0.0232). Following 24 h of drug exposure, pradofloxacin (4.0 log_10_, 99.99% kill) killed more cells than florfenicol (0.6 log_10_, 63.5% kill, *p* < 0.0001), tildipirosin (0.4 log_10_, 42.2% kill, *p* < 0.0001), tilmicosin (0.12 log_10_, *p* < 0.0001), and tulathromycin (0.6 log_10_, *p* < 0.0001). Statistically significant differences were seen for treatment, time, and treatment by time (*p* values from 0.0006–<0.0001) for all comparisons.

#### 3.1.4. Maximum Tissue Drug Concentration (T_issuemax_)

Following exposure of 10^6^ cfu/mL of *P. multocida* to the maximum tissue drug concentrations tested, statistically significant differences in kill rates between the agents were not seen following 30 min and 1 h of drug exposure (Table 2). Pradofloxacin killed 97.2%, 99.8%, and 99.99% of cells following 30 min, 2 h, and 12 h after drug exposure, and 100% after 24 h of drug exposure. Ceftiofur and enrofloxacin killed 55.3% and 75.2%, 60.9% and 98.3%, and 100% each following 30 min, 2 h, and 24 of drug exposure. Florfenicol killed 37.8% of cells following 1 h of drug exposure but growth occurred at all time points thereafter. Tildipirosin killed 3.6% of cells following 30 min of drug exposure, which increased to 25.7% and 99.9% following 2 and 24 h of drug exposure. Tulathromycin killed between 28.4 and 32.5% of cells following 4–6 h of drug exposure but regrowth occurred thereafter. Following 2 h of drug exposure, pradofloxacin (2.8 log_10_, 99.8% kill) killed more cells than tildipirosin (0.2 log_10_, 25.7% kill, *p* = 0.0370). Following 4 h of drug exposure, pradofloxacin (2.8 log_10_, 99.4% kill) killed more cells than florfenicol (growth, *p* = 0.0179). Following 6 h of drug exposure, ceftiofur (2.9 log_10_, 91.1% kill) killed more cells than florfenicol (0.2 log_10_, *p* = 0.0370). Following 12 h of drug exposure, ceftiofur (4.1 log_10_, 99.9% kill) killed more cells than florfenicol (0.4 log_10_, *p* < 0.0001), tildipirosin (1.4 log_10_, *p* = 0.0077), and tulathromycin (2.7 log_10_, *p* = 0.0101). Enrofloxacin (3.2 log_10_, 99.8% kill) killed more cells than florfenicol (*p* = 0.0062). Pradofloxacin (5.1 log_10_, 99.99% kill) killed more cells than florfenicol (*p* < 0.0001), tildipirosin (*p* < 0.0001), and tulathromycin (*p* = 0.0001). Following 24 h of drug exposure, ceftiofur (3.8 log_10_, 100% kill) killed more cells than florfenicol (1.5 log_10_, *p* < 0.0001). Enrofloxacin (6.8 log_10_, 100% kill) killed more cells than florfenicol (*p* < 0.0001) and tildipirosin (3.6 log_10_, 99.9% kill, *p* = 0.0024). Pradofloxacin (6.9 log_10_, 100% kill) killed more cells than florfenicol (*p* < 0.0001) and tildipirosin (3.6 log_10_, 99.9% kill, *p* = 0.0008). Statistically significant differences were seen in treatment (*p* = 0.0001), time (*p* < 0.0001), and treatment by time (*p* < 0.0001).

Exposure of 10^7^ cfu/mL of *P. multocida* to the maximum tissue drug concentrations of the drugs tested did not result in significant differences in kill rates between drugs following 30 min of drug exposure (Table 3). Pradofloxacin killed 96.7%, 99.9%, and 100% of cells following 30 min, 6 h, and 24 h after drug exposure; for enrofloxacin, respectively, the values were 70.4%, 99.8%, and 100%. Ceftiofur and tildipirosin killed 50.7% and 4.1%, 97.7% and 56.2%, and 98.5% and 99.7% following 30 min, 6 h, and 24 h of drug exposure. Florfenicol killed 17.8% and 15.9% of cells following 30 min and 2 h of drug exposure but growth occurred at all other time points. Tulathromycin killed 10.2% and 25.8% of cells following 1–2 h of drug exposure and growth occurred at all other time points. Following 1 and 2 h of drug exposure, pradofloxacin (2.2–2.4 log_10_, 97.8–99.1% kill) killed more cells than florfenicol (0.03 log_10_, *p* = 0.0217, *p* = 0.0068). Following 4 h of drug exposure, enrofloxacin (2.1 log_10_, 99.0% kill) and pradofloxacin (3.6 log_10_, 99.4% kill) killed more cells than florfenicol (growth, *p* = 0.0223 and *p* = 0.0100). Pradofloxacin killed more cells than tildipirosin (0.2 log_10_, 37.7% kill, *p* = 0.0100). Following 6 h of drug exposure, ceftiofur (1.9 log_10_, 97.7% kill), enrofloxacin (2.8 log_10_, 99.8% kill), and pradofloxacin (3.2 log_10_, 99.9% kill) killed more cells than florfenicol (growth, *p* = 0.063, *p* < 0.0001, *p* < 0.0001). Enrofloxacin and pradofloxacin killed more cells than tildipirosin (0.4 log_10_, 56.2% kill, pp = 0.0043 and *p* = 0.0043) and tulathromycin (growth, *p* = 0.0296 and *p* = 0.0282). Following 12 h of drug exposure, ceftiofur (2.2 log_10_, 99.9%, *p* = 0.0015), enrofloxacin (3.8 log_10_, 99.9% kill, *p* < 0.0001), and pradofloxacin (4.6 log_10_, 99.99% kill, *p* < 0.0001) killed more cells than florfenicol (growth). Pradofloxacin killed more cells than ceftiofur (*p* = 0.0342). Enrofloxacin and pradofloxacin killed more cells than tildipirosin (1.2 log_10_, 90.8% kill, *p* = 0.0016 and *p* < 0.0001) and tulathromycin (1.4 log_10_, *p* < 0.0001 and *p* < 0.0001). Following 24 h of drug exposure, enrofloxacin (7.9 log_10_, 100% kill) and pradofloxacin (7.5 log_10_, 100% kill) killed more cells than ceftiofur (2.2 log_10_, 98.5% kill, *p* < 0.0001 and *p* < 0.0001). Enrofloxacin and pradofloxacin killed more cells than florfenicol (0.6 log_10_, *p* < 0.0001 and *p* < 0.0001). Enrofloxacin and pradofloxacin killed more cells than tildipirosin (2.9 log_10_, 99.7% kill, *p* < 0.0001 and *p* < 0.0001) and tulathromycin (1.4 log_10_, *p* < 0.0001 and *p* < 0.0001). Tildipirosin and tulathromycin killed more cells than florfenicol (*p* = 0.0004 and *p* = 0.0068). Statistically significant differences were seen for treatment, time, and treatment by time (*p* < 0.0001) for all comparisons.

Following exposure of 10^8^ cfu/mL of *P. multocida* to the maximum tissue drug concentration of the drugs tested, significant differences in kill rates between the drugs were not seen in the 30 min, 1 h, and 2 h following drug exposure (Table 4). Pradofloxacin killed 97.0%, 99.7%, and 99.99% of cells following 30 min, 4 h, and 24 h of drug exposure; for enrofloxacin, those values were 62.9%, 94.7%, and 97.1%. Maximal killing (47.6%) in the presence of florfenicol occurred following 24 h of drug exposure. Growth occurred at all time points in the presence of tulathromycin. Maximal killing (3.4%) in the presence of tildipirosin occurred following 24 h of drug exposure. Ceftiofur killed 32.9%, 78.1%, and 93.5% of cells following 30 min, 4 h, and 24 h of drug exposure. Following 4 h of drug exposure, pradofloxacin (3.1 log_10_, 99.7% kill) killed more cells than tildipirosin (growth, *p* = 0.0313). Following 12 h of drug exposure, enrofloxacin (2.7 log_10_, 97.7% kill) killed more cells than tulathromycin (growth, *p* = 0.0294) and pradofloxacin (3.6 log_10_, 99.9% kill) killed more cells than tildipirosin (growth, *p* = 0.0019) and tulathromycin (*p* = 0.0001). Pradofloxacin killed more cells than florfenicol (growth, *p* = 0.0144). Following 24 h of drug exposure, pradofloxacin (4.5 log_10_, 99.99% kill) killed more cells than ceftiofur (1.2 log_10_, 93.5% kill, *p* = 0.0066), florfenicol (0.2 log_10_, 47.6% kill, *p* = 0.0001), tildipirosin (0.1 log_10_, 3.4% kill, *p* < 0.0001), and tulathromycin (growth, *p* < 0.0001). Enrofloxacin (2.9 log_10_, 97.1% kill) killed more cells than tulathromycin (*p* = 0.0095). Statistically significant differences were seen for treatment, time, and treatment by time (*p* values from 0.0037–<0.0001) for all comparisons.

Following exposure of 10^9^ cfu/mL of *P. multocida* to the maximum tissue drug concentration of the drugs tested, statistically significant kill rates were not seen between the drugs following 30 min, 1, 2, 4, 6, and 12 h of drug exposure (Table 5). Pradofloxacin killed 73.6%, 94.2%, and 99.9% of cells following 30 min, 4 h, and 24 h of drug exposure; for enrofloxacin, those values were 36.9%, 62.1%, and 96.2%; for ceftiofur, those values were 4.5%, 13.7%, and 89.9%. Maximal killing (57.7%) in the presence of florfenicol occurred following 24 h of drug exposure. Minimal killing (4.4–14.9%) in the presence of tildipirosin and tulathromycin occurred following 30 min to 2 h following drug exposure, but growth occurred at all other time points for both drugs. Following 24 h of drug exposure, pradofloxacin (5.5 log_10_, 99.9% kill) killed more cells than ceftiofur (1.1 log_10_, 89.9% kill, *p* = 0.0004), enrofloxacin (1.6 log_10_, 96.2% kill, *p* = 0.0019), florfenicol (0.5 log_10_, 57.7% kill, *p* < 0.0001), tildipirosin (growth, *p* < 0.0001), and tulathromycin (growth, *p* < 0.0001). Statistically significant differences were seen for treatment, time, and treatment by time (*p* values from 0.0156–<0.0063) for all comparisons.

## 4. Discussion

Swine respiratory disease has an enormous economic impact in the USA and globally, costing the swine industry in excess of a billion dollars annually in the USA alone [22]. *P. multocida* is a well-recognized bacterial pathogen associated with swine respiratory disease and may be found as a co-pathogen with other microorganisms [23]. It has a number of identified virulence factors, including a capsule for avoiding phagocytosis, lipopolysaccharide, surface adhesions, iron acquisition proteins, and the *P. multocida* toxin associated with atrophic rhinitis [24]. Tigga et al. reported on an outbreak of swine pasteurellosis in India [25]. The case fatality rate was 95% in adult animals and 100% in piglets, with death occurring after a clinical course of 4–6 days. Marruchella et al. reported a sow mortality rate of 15% from Brazilian farms, with *P. multocida* being the sole cause following the probable introduction of a “new” strain that subsequently infected immunologically naïve animals [26]. In a comprehensive review of post-weaning mortality in commercial swine production, Gebhard and colleagues, using a 3+ system (+ being low to +++ being high), reported *P. multocida* being 2+ for incidence and 2+ for magnitude of factors contributing to mortality [27]. As such, *P. multocida* is an important swine pathogen and determining antimicrobial activity against this pathogen is of paramount importance for defining drugs active against this organism.

The traditional definition of bacterial versus bacteriostatic is based on differences in log_10_ reduction in bacterial cells in the presence of the drug. For example, a ≥3 log_10_ reduction in bacterial cells defined a bactericidal agent whereas a ≤2 log_10_ reduction defined a bacteriostatic agent with a log_10_ reduction between 2 and 3 being a “grey zone” [28]. Such definitions were based on testing a 10^5^ cfu/mL inocula and may not similarly apply to higher cell density kill measurements, as described here [13].

Another in vitro measurement is the minimal bactericidal concentration (MBC) or the lowest antibiotic concentration that kills a specific proportion (i.e., 99.9%) of the starting bacterial population [29,30]. MBC is determined from broth microdilution MIC assays utilizing 10^5^ cfu/mL. The MBC is usually 1–2 dilutions higher than the MIC and is determined by plating from microtiter plate wells to drug free agar and growth quantified. MPC testing was not utilized in this study, as bacterial densities in excess of 10^5^ cfu/mL were used (e.g., 10^6^–10^9^ cfu/mL).

From this study, we tested antimicrobial agents classified as bacteriostatic and bactericidal based on the definitions provided above. Pankey and Sabath questioned the clinical relevance of bacteriostatic versus bactericidal designations and that in vitro observations need to be correlated with in vivo effects [31]. Finberg et al. indicated that bactericidal agents are important for treating endocarditis and meningitis but also indicated that a drug classified as bacteriostatic against one organism many exhibit bactericidal activity against a different organisms and drug concentrations may also influence static versus bactericidal in vitro activity [32]. Lobritz and colleagues reported antibiotic efficacy was linked to bacterial cellular respiration [33]. They found bacteriostatic antibiotics decelerate cellular respiration and thereby generate a metabolic state that is prohibitive to killing. When bacteriostatic agents are used in combination with bactericidal drugs, a bacteriostatic agent can block bactericidal lethality. Baquero and Levin commented on the various factors influencing bactericidal activity of antibiotics with susceptible bacteria [34]. These include free drug, as only free drug is active, and protein binding (higher levels of protein binding is often seen with beta lactam agents) reduces the level of active drug, uptake systems allowing for or preventing drug entry to cells, target binding affinity, targeted function, cellular repair of damaged functions, activated inducible antibiotic-deactivating mechanisms, alternative functions to bypass those that are inhibited, induction of reactive oxygen species, low replication rates, and others.

Pradofloxacin is the newest approved veterinary fluoroquinolone, having recently has been approved for use in food animals, including swine with swine respiratory disease. Fluoroquinolones kill bacterial cells by inhibiting two enzymes critical for DNA replication—namely, DNA gyrase (topoisomerase II) and topoisomerase IV. Enrofloxacin and marbofloxacin preferentially target DNA gyrase in Gram-negative bacteria whereas in Gram-positive organisms, topoisomerase IV is the primary enzyme target. Pradofloxacin simultaneously targets both DNA gyrase and topoisomerase IV and as such two simultaneous mutations would be required for resistance to pradofloxacin to occur [35].

In a previous study from our laboratory on bovine respiratory pathogens, including *P. multocida*, we tested the same antibiotics as reported here [36]. The bacterial density in the kill assays in that report was 10^5^ cfu/mL. Following exposure to the drug MIC, pradofloxacin and enrofloxacin killed 95% of cells following 180 min of drug exposure as compared to growth—73% kill with the other agents. Exposure to the MPC drug concentration resulted in 94–99% kill following 180 min of drug exposure for all agents except ceftiofur (9.7% kill) and florfenicol (41% kill). At the maximum serum drug concentration and following 60 min of drug exposure, pradofloxacin killed 94% of cells (growth—66.7% kill for comparators), and following 180 min of drug exposure, pradofloxacin killed 99.3% of cells (growth to 96.% kill for comparators). Finally, at the maximum tissue drug concentrations, pradofloxacin killed 99% of cells following 180 min of drug exposure (89.7–98.4% kill for comparators). In this study, killing was measured against a range of bacterial densities from 10^6^–10^9^ cfu/mL, as high bacterial densities are documented in acute infections [6,7,8,9,10,11,37] and are known to fluctuate during infection. Bioburden has been linked to risk for infection progression and severity [38,39].

In this study and against a range of bacterial densities tested, pradofloxacin killed between 96 and 99% of bacterial cells following 4–24 h of drug exposure at the drug MPC against the 10^6^–10^8^ cfu/mL bacterial densities; 59–83% of cells were killed following 4–24 h of drug exposure against the 10^9^ cfu/mL bacterial density. Faster and more extensive killing occurred with shorter drug exposure at the maximum serum and tissue drug concentrations. For example, following exposure to the maximum serum drug concentration, 96–>99% of cells were killed following 30 min to 4 h of drug exposure with similar observations at the maximum tissue drug concentration. Similarly, enrofloxacin and marbofloxacin exhibited bactericidal activity, killing 98–100% of cells at the maximum serum drug concentration following 12–24 h of drug exposure. Ceftiofur killed 92–99.9% of cells following 24 h of drug exposure at the maximum serum concentration. The killing by florfenicol was variable but at the maximum serum drug concentration killed 95.9–99.9% of cells at the 10^6^–10^7^ cfu/mL density but less at the 10^8^–10^9^ cfu/mL densities. Finally, tildipirosin, tilmicosin, and tulathromycin were bacteriostatic, killing minimal bacterial cells or having growth in the presence of the drug over the higher bacterial densities tested.

In vitro studies such as this have limitations that need to be highlighted. First, an in vitro measurement cannot duplicate the interaction that occurs in infected animals where immune responses occur. Second, serum and tissue drug concentration used in this study may vary depending on how these values were determined and potentially influenced by collection methods. Third, we use drug concentrations that remain constant over the duration of the experiments and do not simulate drug elimination over time as occurs in treated animals. Four, the kill experiments are based on tested drug and not corrected for protein binding, which varies considerably between the agents tested. Despite the above limitations, this study provides a standardized comparison of agents for killing *P. multocida* strains in controlled experiments and shows differences in the speed and extent of killing. How these observations relate clinically need to be investigated in vivo.

## 5. Conclusions

In summary, pradofloxacin is the most recent veterinary fluoroquinolone approved in the USA for treating swine respiratory disease. It is rapidly bactericidal and the dual enzyme targeting mechanism of action reduces the likelihood of resistance selection from susceptible bacterial populations. Pradofloxacin appears to be an important addition to available drugs used for treating bacterial infections in pigs.

## Figures and Tables

**Table 1:** Comparative MIC, MPC, and therapeutic drug concentration values for 8 antimicrobial agents.

	Isolates						C_max_ (µg/mL)	T_issuemax_ (µg/mL)
	#5		#6		#14			
*P. multocida*	MIC	MPC	MIC	MPC	MIC	MPC		
Ceftiofur	0.002	0.125	0.002	0.125	0.002	0.25	6.9	2.64
Enrofloxacin	0.008	0.063	0.004	0.063	0.008	0.031	1.9	4.6
Florfenicol	0.5	1	0.25	1	0.5	0.5	4.5	2.94
Marbofloxacin	0.016	0.125	0.008	0.125	0.016	0.25	1.5	NT
Pradofloxacin	≤0.008	0.031	0.031	0.031	0.004	0.25	2.64	0.81
Tildipirosin	1	4	0.5	4	0.5	4	0.767	14.77
Tilmicosin	4	32	2	8	2	4	0.25	NT
Tulathromycin	0.5	2	0.25	1	0.5	1	0.6	3.2

**Table 2:** Log_10_ reduction in viable cells and percentage of cells killed over time to suspension of *P. multocida* (10^6^) exposed to various concentrations of 8 drugs.

Minimum Inhibitory Concentration																
Variable	Ceftiofur		Enrofloxacin		Florfenicol		Marbofloxacin		Pradofloxacin		Tildipirosin		Tilmicosin		Tulathromycin	
(h)	Log_10_	%Kill	Log_10_	%Kill	Log_10_	%Kill	Log_10_	%Kill	Log_10_	%Kill	Log_10_	%Kill	Log_10_	%Kill	Log_10_	%Kill
0.5	−0.07	−15.77	0.09	24.40	0.03	8.45	−0.03	−6.16	−0.26	−23.13	0.06	14.56	0.19	356.12	−0.04	−7.26
1	−0.24	−36.99	−0.10	−18.80	0.08	21.59	0.01	2.44	−0.37	−11.21	0.07	17.90	0.05	20.29	−0.01	2.02
2	−0.29	−29.98	0.06	37.85	0.14	40.45	−0.05	−8.75	−0.35	−26.85	0.09	32.52	0.21	129.98	0.04	32.89
4	−0.60	−63.27	−0.10	−0.91	0.16	823.77	−1.04	−90.46	−1.68	−97.68	−0.35	−49.07	−0.01	375.68	0.11	221.34
6	−0.70	−72.15	−0.30	−31.64	−0.30	42.73	−1.44	−95.83	−1.67	−96.92	−0.20	−34.76	0.14	354.96	0.20	258.02
12	−0.41	−46.26	0.35	312.61	−0.40	41.97	−1.59	−96.81	−1.86	−98.14	−1.36	−88.11	0.58	395.17	0.31	476.62
24	−0.09	255.16	0.33	343.48	−0.08	921.93	−2.45	−99.58	−4.07	−98.84	−1.19	−90.54	0.47	363.90	0.17	474.17
Mutant Prevention Concentration																
0.5	−0.05	−8.60	0.07	18.62	0.08	31.51	−0.71	−41.91	−0.50	−56.72	−0.07	−1.03	−0.21	−31.44	0.07	19.44
1	−0.05	−10.23	−0.31	−42.09	0.09	41.65	−1.09	−55.31	−0.90	−79.43	0.01	11.63	−0.09	−38.56	0.03	7.56
2	−0.22	−33.25	−1.02	−73.85	0.15	30.80	−1.45	−72.52	−1.16	−92.93	−0.15	−23.43	−0.81	−53.87	−0.37	−53.28
4	−0.80	−75.79	−1.39	−89.45	−0.39	−41.91	−1.76	−90.96	−1.54	−96.92	−0.23	−32.71	−1.18	−75.66	−0.85	−82.57
6	−1.82	−97.69	−1.61	−96.23	−0.47	−53.84	−2.16	−98.76	−1.76	−96.79	−0.02	7.34	−1.17	−80.55	−1.40	−92.72
12	−2.27	−94.54	−1.74	−88.75	−0.38	−40.90	−2.51	−97.18	−2.91	−99.81	−1.28	−95.92	−1.85	−95.27	−1.61	−95.26
24	−3.71	−99.63	−0.84	−70.43	−0.91	456.46	−4.81	−99.79	−5.24	−99.98	−3.30	−99.92	−4.02	−99.86	−2.34	−99.44
Maximum Serum Drug Concentration																
0.5	−0.55	−55.57	−0.98	−81.67	0.08	19.87	−2.13	−98.95	−1.98	−98.78	−0.03	−3.79	0.13	43.46	0.00	1.05
1	−0.99	−82.44	−1.17	−85.29	−0.08	−14.25	−2.49	−98.95	−1.96	−98.90	0.06	21.92	0.16	47.43	0.06	15.31
2	−1.24	−86.66	−1.54	−95.76	−0.17	−16.70	−2.59	−99.26	−2.20	−99.22	0.03	24.01	0.40	225.52	0.22	98.10
4	−2.00	−97.66	−2.13	−98.65	−0.97	−83.82	−2.63	−99.42	−3.42	−99.94	−0.27	−37.14	0.78	677.30	0.12	138.71
6	−2.78	−99.66	−2.43	−99.61	−0.89	−85.49	−2.74	−99.89	−3.90	−99.97	−0.28	−40.51	0.98	950.71	−0.12	141.33
12	−4.33	−99.98	−3.63	−99.95	−1.14	−79.55	−3.32	−99.80	−5.93	−100.00	−2.45	−99.30	1.03	1062.11	−0.57	−66.48
24	−5.43	−99.99	−6.62	−100.00	−4.01	−99.87	−6.45	−99.95	−6.74	−100.00	−4.91	−99.99	1.03	1062.11	−1.89	−93.80
Maximum Tissue Drug Concentration																
0.5	−0.38	−55.30	−0.86	−75.15	−0.04	1.78			−1.99	−97.22	−0.02	−3.56			−0.08	−14.82
1	−0.46	−52.03	−1.37	−93.76	−0.25	−37.80			−2.20	−96.84	0.00	−0.96			−0.11	−19.83
2	−0.63	−60.88	−1.79	−98.29	−0.10	0.17			−2.78	−99.80	−0.16	−25.69			−0.26	−32.51
4	−2.01	−98.05	−2.05	−98.63	0.15	197.83			−2.76	−99.41	−0.26	−40.42			−0.29	−28.36
6	−2.96	−91.13	−2.57	−99.64	−0.16	423.63			−2.64	−99.27	−0.45	−51.08			−0.32	−7.24
12	−4.10	−99.94	−3.15	−99.82	−0.35	417.89			−5.06	−99.99	−1.38	−93.05			−0.69	197.81
24	−3.79	−100.00	−6.75	−100.00	−1.53	1900.05			−6.88	−100.00	−3.62	−99.93			−3.33	194.99

**Table 3:** Log_10_ reduction in viable cells and percentage of cells killed over time to suspension of *P. multocida* (10^7^) exposed to various concentrations of 8 drugs.

Minimum Inhibitory Concentration																
Variable	Ceftiofur		Enrofloxacin		Florfenicol		Marbofloxacin		Pradofloxacin		Tildipirosin		Tilmicosin		Tulathromycin	
(h)	Log_10_	%Kill	Log_10_	%Kill	Log_10_	%Kill	Log_10_	%Kill	Log_10_	%Kill	Log_10_	%Kill	Log_10_	%Kill	Log_10_	%Kill
0.5	0.00	1.20	0.22	70.61	0.03	11.40	−0.03	4.23	−0.26	27.20	0.06	61.80	0.19	6.79	−0.04	19.51
1	−0.10	−7.25	0.22	67.21	0.08	15.29	0.01	7.53	−0.37	−58.49	0.07	74.28	0.05	17.52	−0.01	4.80
2	−0.16	−21.81	0.19	103.15	0.14	36.46	−0.05	9.62	−0.35	−17.33	0.09	75.12	0.21	60.68	0.04	62.12
4	−0.11	−21.17	0.40	156.71	0.16	157.27	−1.04	22.11	−1.68	0.54	−0.35	172.37	−0.01	85.26	0.11	183.05
6	−0.50	−49.35	0.54	275.81	−0.30	310.00	−1.44	135.01	−1.67	37.29	−0.20	461.99	0.14	391.80	0.20	210.39
12	−0.31	4.32	0.91	747.13	−0.40	1217.29	−1.59	522.56	−1.86	−87.83	−1.36	1046.11	0.58	220.13	0.31	816.10
24	0.10	70.73	0.96	1073.33	−0.08	1095.23	−2.45	711.44	−4.07	128.81	−1.19	354.70	0.47	156.18	0.17	846.74
Mutant Prevention Concentration																
0.5	−0.03	−5.57	−0.15	−27.46	0.14	40.49	−0.80	−65.26	−0.70	−65.04	−0.03	−5.72	−0.06	−11.12	0.02	6.78
1	−0.07	−13.31	−0.48	−54.16	0.09	28.29	−0.80	−68.10	−1.01	−84.45	0.02	5.82	−0.45	−36.91	0.11	30.57
2	0.00	−29.91	−0.98	−68.33	0.15	42.35	−1.19	−79.34	−1.02	−88.35	−0.17	−28.49	−0.75	−32.03	0.03	11.89
4	−0.59	−72.21	−1.26	−77.64	0.10	547.76	−1.50	−45.95	−1.69	−97.89	−0.37	−56.32	−0.95	−64.31	−0.45	−39.70
6	−0.77	−71.83	−1.16	−24.02	0.20	80.21	−1.46	−64.80	−2.10	−98.69	−0.05	−4.58	−1.06	−25.59	−0.56	−30.87
12	−0.95	−84.22	−1.12	21.85	0.50	307.83	−1.78	−33.57	−2.78	−99.67	−1.12	−92.22	−1.08	−49.30	−0.41	−15.05
24	−1.01	−89.74	−1.37	270.56	0.40	712.04	−2.35	−14.24	−4.63	−99.98	−2.52	−99.59	−2.15	−61.74	−1.04	−36.88
Maximum Serum Drug Concentration																
0.5	−0.42	−30.80	−1.09	−83.43	0.02	6.27	−1.99	−98.48	−2.04	−98.98	−0.07	−6.66	0.02	5.50	0.09	23.12
1	−1.19	−81.44	−1.24	−90.34	0.05	15.74	−2.37	−99.10	−1.91	−98.74	0.09	26.10	0.01	3.92	0.12	34.68
2	−1.19	−78.82	−1.69	−95.19	−0.12	−21.55	−2.58	−99.31	−1.87	−96.10	−0.22	−17.87	0.16	52.35	0.25	103.91
4	−1.52	−94.32	−2.03	−99.45	−0.38	−51.72	−2.72	−99.35	−2.78	−99.77	−0.04	21.33	0.52	276.03	0.45	266.09
6	−1.26	−93.06	−2.22	−99.08	−0.29	−41.50	−2.94	−99.74	−2.99	−99.82	0.11	32.11	0.55	270.75	0.57	329.77
12	−1.54	−93.52	−2.90	−99.75	−0.65	−75.84	−4.67	−99.99	−4.92	−99.96	−0.26	−13.11	0.91	770.45	1.03	997.92
24	−2.20	−92.38	−6.60	−33.33	−1.72	−95.86	−7.02	−100.00	−7.81	−100.00	−0.88	45.81	0.92	792.94	1.17	1555.22
Maximum Tissue Drug Concentration																
0.5	−0.07	−50.69	−0.92	−70.43	−0.13	−17.84			−1.89	−96.71	−0.02	−4.07			0.01	2.75
1	−0.94	−72.38	−1.33	−92.62	−0.03	5.81			−2.17	−97.79	0.01	4.37			−0.05	−10.22
2	−1.07	−85.08	−1.73	−97.51	−0.13	−15.88			−2.43	−99.09	−0.13	−16.79			−0.22	−25.82
4	−1.73	−96.13	−2.08	−99.04	0.02	69.90			−3.55	−99.41	−0.23	−37.71			−0.18	89.30
6	−1.95	−97.74	−2.83	−99.80	0.20	292.88			−3.21	−99.91	−0.40	−56.21			0.00	104.90
12	−2.19	−99.03	−3.77	−99.93	0.16	382.29			−4.64	−99.99	−1.20	−90.77			−0.15	381.62
24	−2.21	−98.53	−7.91	−100.00	−0.61	56.00			−7.51	−100.00	−2.97	−99.71			−1.42	205.60

**Table 4:** Log_10_ reduction in viable cells and percentage of cells killed over time to suspension of *P. multocida* (10^8^) exposed to various concentrations of 8 drugs.

Minimum Inhibitory Concentration																
Variable	Ceftiofur		Enrofloxacin		Florfenicol		Marbofloxacin		Pradofloxacin		Tildipirosin		Tilmicosin		Tulathromycin	
(h)	Log_10_	%Kill	Log_10_	%Kill	Log_10_	%Kill	Log_10_	%Kill	Log_10_	%Kill	Log_10_	%Kill	Log_10_	%Kill	Log_10_	%Kill
0.5	−0.07	−14.90	0.06	21.55	−0.04	−5.40	0.00	0.45	−0.57	−43.56	0.08	21.96	0.02	10.19	0.13	36.25
1	−0.14	−26.92	0.19	59.35	0.16	282.75	0.08	23.51	−0.76	−57.33	0.03	8.21	−0.03	−6.54	0.17	47.58
2	−0.04	2.10	0.13	45.94	0.17	48.88	0.00	10.62	−0.86	−42.99	0.09	23.85	0.10	27.90	0.22	64.71
4	−0.05	−9.26	0.20	60.90	0.10	32.45	0.08	36.54	−1.39	−60.48	0.17	50.24	−0.03	9.13	0.32	111.32
6	−0.01	2.40	0.14	45.17	0.16	58.89	0.03	64.46	−1.12	−20.00	0.35	128.50	−0.07	−8.57	0.26	83.35
12	−0.06	−11.77	0.25	76.91	0.24	79.49	0.12	234.78	−0.87	−34.12	0.43	212.97	0.13	53.31	0.43	182.41
24	0.03	23.56	0.43	367.39	0.05	31.05	0.21	206.47	−1.56	−15.33	0.29	139.76	−0.04	−7.54	0.16	51.04
Mutant Prevention Concentration																
0.5	0.15	43.19	0.00	0.86	−0.25	38.22	−0.67	−63.73	−0.68	−65.85	0.02	11.05	−0.24	−31.76	0.05	14.04
1	0.07	51.57	−0.33	−48.02	0.13	61.78	−0.85	−62.41	−1.00	−82.79	0.01	10.20	−0.41	−43.74	0.08	31.96
2	0.01	2.38	−0.46	−47.26	0.16	90.54	−0.67	−72.17	−1.34	−93.14	−0.07	−13.26	−0.46	−10.88	0.12	24.40
4	−0.29	−43.47	−0.58	−39.01	0.16	49.46	−0.85	−51.34	−1.70	−96.31	−0.11	−20.80	−0.47	−25.35	0.27	132.81
6	−0.33	−47.53	−0.63	−31.72	0.26	93.26	−1.33	−52.19	−1.73	−97.24	0.18	51.80	−0.60	−25.05	0.32	110.24
12	−0.33	39.36	−0.26	0.46	0.77	1289.76	−1.37	−45.43	−1.37	−93.41	−0.05	−2.03	−0.35	49.77	0.31	92.90
24	−0.15	−25.54	−0.08	191.34	0.42	181.70	−1.53	−36.87	−2.08	−96.99	−0.16	−30.33	−0.80	−37.77	0.42	741.93
Maximum Serum Drug Concentration																
0.5	−0.39	−55.53	−0.83	−66.08	−0.04	−9.12	−1.90	−98.39	−1.96	−98.59	−0.16	−28.68	0.07	22.32	0.04	6.41
1	−0.77	−85.19	−1.03	−82.83	0.02	4.31	−2.29	−99.12	−1.87	−98.90	−0.01	3.35	0.04	10.27	0.12	29.51
2	−1.07	−90.82	−1.61	−89.92	0.05	12.87	−2.63	−99.51	−2.46	−99.57	0.03	9.39	0.08	20.09	0.23	67.17
4	−1.31	−93.09	−2.17	−96.11	0.02	4.39	−3.46	−99.90	−3.30	−99.93	0.28	94.61	0.15	53.05	0.31	99.44
6	−0.89	−85.23	−2.24	−97.55	0.05	13.53	−3.15	−99.86	−3.40	−99.94	0.35	136.10	0.17	57.92	0.24	81.37
12	−0.93	−84.99	−2.49	−97.10	0.29	98.10	−3.43	−99.92	−5.84	−99.99	0.02	−47.82	0.33	117.39	0.22	62.95
24	−1.66	−96.04	−3.18	−99.79	−0.02	2.08	−4.14	−99.98	−5.90	−99.99	−0.08	−16.68	0.53	554.00	0.30	136.14
Maximum Tissue Drug Concentration																
0.5	−0.21	−32.86	−0.91	−62.89	−0.08	−13.54			−1.98	−97.00	0.01	3.18			0.06	13.62
1	−0.80	−70.42	−1.31	−87.02	−0.07	−33.74			−2.26	−97.97	0.01	4.47			0.09	23.60
2	−0.63	−72.00	−1.83	−96.82	−0.03	−6.16			−2.92	−99.30	0.04	9.10			0.12	31.69
4	−0.79	−78.05	−2.10	−94.74	−0.06	−8.24			−3.08	−99.69	0.02	6.12			0.23	75.56
6	−1.04	−86.48	−2.32	−97.70	0.13	11.57			−2.90	−99.75	0.06	15.79			0.24	82.81
12	−0.98	−87.93	−2.69	−97.73	0.22	69.31			−3.57	−99.96	0.00	17.86			0.58	326.63
24	−1.21	−93.52	−2.94	−97.11	−0.19	−47.56			−4.51	−99.99	−0.06	−3.38			0.32	162.79

**Table 5:** Log_10_ reduction in viable cells and percentage of cells killed over time to suspension of *P. multocida* (10^9^) exposed to various concentrations of 8 drugs.

Minimum Inhibitory Concentration																
Variable	Ceftiofur		Enrofloxacin		Florfenicol		Marbofloxacin		Pradofloxacin		Tildipirosin		Tilmicosin		Tulathromycin	
(h)	Log_10_	%Kill	Log_10_	%Kill	Log_10_	%Kill	Log_10_	%Kill	Log_10_	%Kill	Log_10_	%Kill	Log_10_	%Kill	Log_10_	%Kill
0.5	−0.04	−8.44	0.03	9.73	0.05	11.79	−0.03	−2.82	−0.17	19.26	0.01	6.20	−0.02	−2.88	0.10	30.29
1	−0.02	−4.26	−0.02	−3.52	0.12	30.19	0.04	10.89	−0.34	−9.01	−0.01	−0.93	0.02	19.61	0.16	47.29
2	−0.01	−1.41	−0.05	−17.01	0.07	53.02	0.05	13.97	−0.21	62.18	0.02	6.75	0.05	25.19	0.12	31.96
4	0.06	17.45	0.04	9.19	−0.06	−10.87	0.02	6.93	−0.50	−11.95	0.03	8.44	−0.08	3.52	0.13	37.96
6	0.02	41.58	0.06	16.82	−0.07	−7.00	0.08	22.54	−0.59	17.60	−0.17	−14.11	−0.12	−11.07	0.15	43.48
12	0.01	6.05	0.05	13.22	0.10	36.90	0.26	94.33	0.10	34.00	0.09	31.84	0.18	73.47	−0.07	1.02
24	−0.24	23.96	−0.13	−23.36	−0.17	−29.72	0.11	50.33	−0.84	−6.19	−0.10	−13.03	0.10	−31.99	−0.30	−20.79
Mutant Prevention Concentration																
0.5	−0.03	−2.86	0.35	116.77	−0.02	2.30	−0.09	−18.77	−0.62	−60.86	0.11	27.58	−0.11	−6.07	0.14	52.85
1	−0.08	−13.23	0.08	22.77	0.06	15.67	−0.16	−30.72	−0.71	−66.46	0.14	30.19	−0.11	−2.27	0.19	72.51
2	−0.13	−16.22	0.17	49.26	0.04	11.24	−0.18	−30.21	−0.80	−67.12	0.13	34.09	−0.39	−51.59	0.06	35.10
4	−0.21	−36.96	0.09	24.78	−0.11	−21.45	−0.18	−31.87	−0.96	−59.96	0.08	16.25	−0.17	14.18	0.09	34.22
6	−0.23	−40.27	0.04	9.36	−0.05	−10.71	−0.21	−37.83	−1.25	−75.20	0.24	57.93	−0.50	−58.44	0.06	26.06
12	−0.31	−49.35	0.12	33.36	0.15	55.77	−0.07	−11.67	−0.61	−43.76	0.07	27.78	−0.20	−10.28	0.24	101.66
24	−1.23	−78.83	0.37	287.36	−0.26	−8.81	−0.28	−20.56	−1.69	−83.35	−0.40	−44.82	−0.39	−38.62	0.31	910.46
Maximum Serum Drug Concentration																
0.5	−0.05	−7.85	−0.84	−84.96	−0.01	−0.92	−1.40	−95.71	−1.55	−96.90	−0.10	−18.51	0.07	23.42	0.12	37.45
1	−0.13	−25.19	−1.08	−90.48	−0.08	−14.65	−1.80	−98.04	−1.62	−97.48	0.10	31.36	0.17	57.80	0.03	16.77
2	−0.07	−15.95	−1.27	−91.08	−0.04	−5.74	−1.85	−98.47	−2.07	−98.91	−0.02	−0.62	0.02	10.17	0.06	15.83
4	−0.16	−27.27	−1.80	−98.21	−0.42	−23.73	−1.97	−98.35	−2.26	−99.26	−0.11	−21.31	0.07	−9.40	−0.10	−16.09
6	−0.19	−35.71	−1.57	−97.38	−0.04	−8.19	−1.83	−95.94	−2.59	−99.69	0.07	20.53	0.07	19.48	0.00	3.91
12	−0.34	−47.03	−1.41	−95.23	0.24	75.05	−1.92	−97.34	−3.04	−99.90	0.05	23.44	0.14	41.72	−0.36	−46.34
24	−1.62	−95.64	−2.30	−98.59	−0.58	−63.49	−2.28	−98.91	−4.02	−99.99	−0.35	−42.16	−0.12	311.60	−0.60	81.45
Maximum Tissue Drug Concentration																
0.5	−0.15	−4.51	−0.36	−36.96	−0.06	−1.21			−0.73	−73.57	−0.05	−9.70			−0.11	−14.85
1	−0.07	−42.76	−0.49	−44.66	0.21	56.50			−0.99	−89.05	0.03	9.50			−0.05	−4.44
2	0.07	15.31	−0.96	−49.91	−0.13	−22.35			−1.13	−91.31	0.02	4.38			−0.04	−7.10
4	−0.10	−13.68	−0.92	−62.06	−0.26	−37.81			−1.33	−94.18	0.02	6.73			0.15	54.48
6	−0.32	−42.80	−1.00	−58.04	−0.02	13.42			−1.54	−94.92	0.05	13.98			0.11	33.72
12	−0.40	−55.22	−1.15	−71.43	0.22	81.60			−1.59	−96.98	−0.08	12.09			0.35	130.47
24	−1.07	−89.91	−1.59	−96.23	−0.49	−57.67			−5.48	−99.95	−0.01	8.33			0.20	68.68

## Data Availability

The data that support the findings of this study are available from the corresponding author upon reasonable request.
